# Do Parent Mental Illness and Family Living Arrangement Moderate the Effects of the Aussie Optimism Program on Depression and Anxiety in Children?

**DOI:** 10.3389/fpsyt.2018.00183

**Published:** 2018-06-12

**Authors:** Maryanne Cheng, Rosanna M. Rooney, Robert T. Kane, Sharinaz Hassan, Natalie Baughman

**Affiliations:** Faculty of Health Sciences, School of Psychology, Curtin University, Perth, WA, Australia

**Keywords:** prevention programs, child mental health, family context, depression, anxiety

## Abstract

Parent mental illness and family living arrangement are associated with depression and anxiety in children, and may influence the effects of programs that aim to prevent these disorders. This study investigated whether these family context factors moderated the intervention effects of the enhanced Aussie Optimism Positive Thinking Skills program on depression and anxiety in primary school children. The intervention was a universal, cognitive-behavioral program, with a one hour session each week for 10 weeks, delivered by trained teachers. The participants were 502 children from 13 private schools, aged 9–11, with 347 in the intervention group and 155 in the control group. There were 267 females and 235 males. Data from 502 parents was also included. A cluster randomized controlled trial design was used, including eight intervention schools and five control schools. Depression and anxiety were assessed at pre-test, post-test, and 6-months follow-up. Information on parent mental illness and family living arrangement was collected through a parent questionnaire. The data was analyzed using covariance analysis with Generalized Linear Mixed Methods. At baseline, depressive and anxiety symptoms did not differ significantly based on parent mental illness. Symptoms of depression at baseline were significantly higher for children from a higher-risk family living arrangement, but anxiety symptoms were not. Parent mental illness and family living arrangement did not moderate the effects of the program on depression and anxiety at post-test or 6-months follow-up. Parent mental illness moderated the intervention effects on negative self-esteem, an aspect of depression, at post-test, with improvements seen only for children who did not have a parent with a mental illness. The findings indicate an association between family living arrangement and depressive symptoms in children. The findings suggest that the program is effective for children regardless of parent mental illness or family living arrangement, although parent mental illness has the capacity to influence the program's outcomes.

## Introduction

### Depression and anxiety in children and adolescents

Depressive and anxiety disorders are among the most commonly occurring mental illnesses in children and adolescents, with the results of meta-analytic research indicating worldwide prevalence rates of 6.5% for anxiety disorders and 2.6% for depressive disorders (1). Australian prevalence rates are comparable to these, with a recent survey finding that 6.9% of Australian children and adolescents had an anxiety disorder in the past year, and 2.8% had major depressive disorder (2). Depressive and anxiety disorders have an impact on the everyday functioning of children and adolescents across multiple areas of their lives, affecting relationships with family and peers, as well as school attendance and performance (2). The symptoms of depression and anxiety cause considerable distress to individuals experiencing these disorders, whether through the low mood and feelings of hopelessness present for those with depression, or the persistent worrying thoughts that are characteristic of anxiety disorders (3). Depression is a major risk factor for suicidality in young people (4). In Australia, mental disorders have the highest contribution to the burden of disease during late childhood and adolescence, and well into adulthood (5).

Research suggests that the trajectories leading to depressive and anxiety disorders begin in early childhood, highlighting the importance of early prevention (6). Episodes of these disorders during childhood also increase the risk of mental health problems later in life (3, 7). High rates of comorbidity between depressive and anxiety disorders, which have been found to range from 16 to 62% (8), suggest that prevention efforts should target both disorders concurrently (9). The detrimental impacts of these disorders on young people, as well as their capacity to contribute to future mental health problems, highlight the importance of prevention efforts during childhood (10).

### Ecological systems theory and the family context

An understanding of protective factors and risk factors can guide the development and implementation of programs that aim to prevent the onset of depressive and anxiety disorders. Protective and risk factors can be present at an individual, family, or community level (3). Family level factors are of particular importance during childhood (11), which will be further explained using the framework of ecological systems theory (12).

Ecological systems theory presents individuals as existing within multiple social contexts, which have differing levels of influence on them (12). According to Bronfenbrenner's (12) theory, each person is situated within systems, which range from being closer to further from the individual. The systems are the microsystem, mesosystem, exosystem, and macrosystem (12). The microsystem contains those social contexts that are most influential on the individual, which, during childhood, are family and school (3). The interaction between contexts within the microsystem is referred to as the mesosystem, so in this case, the interaction between a child's school and family environments. Considering the joint influence of school and family contexts on the mental health of children, it makes sense to consider both in the prevention of depression and anxiety.

Within the family context, a major risk factor for children developing depressive and anxiety disorders is family history (11, 13). There is increasing evidence that prevention needs to take into account the high risk of developing a mental illness for children who have a parent with a depressive disorder (14). In Australia, 21% of children live in a family where at least one parent has a mental illness (Australian Institute of Health and Welfare; 15). Between 25 and 50% of children who have grown up with a parent who has a mental health problem will have a mental disorder themselves at some point in their lives (15). Parent mental illness presents a risk for children developing a depressive or anxiety disorder through both genetic and environmental contributions (3). Parent mental illness has the capacity to influence the family environment, decreasing stability in the home (16) and affecting parents' abilities to provide support and care for their children (17). Behaviors and thinking styles characteristic of the psychopathology of depressive and anxiety disorders may also pose a risk to children through parental modeling (11, 18). A qualitative study found that parents with anxiety and depressive disorders were generally unaware of the possible impact of their mental health on their children and did not know that they could seek help for their children (19).

Family living arrangement is another factor that has been associated with the mental health of children and adolescents (15). In Australia, 74.7% of children live with biological or adopted parents, 8.1% with stepfamilies or blended families, 17% in single parent families, and less than 1% with grandparents or foster parents (15). Children living with alternate carers other than parents, particularly in the child welfare system, have a higher risk of mental illness, perhaps due to the circumstances that led to this (15). Studies of Australian data have found higher rates of mental disorders among children in stepfamilies, blended families or single parent families, as compared to children living with both original parents (2, 20, 21). This is due to a combination of associated factors, including the stress and financial difficulties involved in separation and single parenthood (21). Exposure to parental conflict is also a risk factor for child internalizing problems (11) and is one of the strongest contributors to child mental health difficulties in separated families (20). Socioeconomic status and parent mental health tend to decrease after parent separation, which influences children's mental health (20). It is not the family living arrangement itself that is a risk factor, but the range of associated factors (20).

### Prevention programs and the school context

Childhood is an optimal time for psychological intervention and prevention (6). Many protective and risk factors, such as social skills and thinking styles, are in a developmental stage during the childhood years (22), and can be targeted in order to decrease the risk of a child developing a mental disorder (23). The majority of prevention programs are run within schools, as they are the best place to reach a large number of children (24), and as previously discussed, are an influential social context for children (3).

Universal programs are provided to the whole population, for example, all students in a school (25). Targeted programs include individuals who are already at risk of developing a disorder, due to personal or contextual factors, or existing symptoms (25). In research, a prevention effect has occurred when there is a lower rate of onset of a psychological disorder in the intervention group, compared to a control group (26). Prevention programs can also display intervention effects, which refer to the reduction in symptoms of the disorder (25). Generally, effect sizes are larger for targeted than universal programs (24, 25, 27, 28). However, this does not mean that universal programs are less effective, as they provide the intervention to the whole population, which includes at-risk individuals (29).

At the current time, the prevention field shows increasing promise, but there is still more scope for improvement (10). Across studies, programs appear able to delay the onset and reduce the symptoms of depressive and anxiety disorders for up to 12 months, after which the effects seem to decrease (27). There is limited evidence of programs that are successful in preventing both depressive and anxiety disorders, even though high comorbidity rates suggest that it is ideal to target both disorders together (29).

### The aussie optimism-positive thinking skills program

The Aussie Optimism Positive Thinking Skills (AO-PTS) program is a universal, cognitive-behavioral program, which draws on the work of psychologists such as Martin Seligman, Aaron Beck, and Albert Ellis (30). It aims to prevent depression and anxiety in children, and to promote social-emotional skills (30). Randomized controlled trials of the program have found that it has been able to reduce depressive symptomology from pre-test to post-test (31, 32), as well as the prevalence of depressive disorder at 9-months follow-up (31). However, no effects were found for student-reported anxiety (33). The program also contributed to a significant decrease in parent-reported emotional difficulties, which included depressive and anxiety symptoms, at post-test and 6-months follow-up (32). The AO-PTS program appears to have short to medium term effects (34), making it comparable to current promising programs, which on average demonstrate medium term effects (10).

An enhanced version of the AO-PTS program has been developed with the goal of increasing the effectiveness of the program (35). This was necessary because the program was not able to reduce anxiety symptoms, and did not have long-term effects. More content on emotions was included, and the cognitive restructuring component was simplified to to make it more developmentally appropriate for 9–11 year olds. A randomized controlled trial on the enhanced AO-PTS program found that the program was effective at preventing symptoms of depression at post-test and 6-months follow-up, and anxiety at 6-months follow-up (Rooney et al., in preparation).

### Moderation effects in prevention programs

Prevention research focuses mainly on between-group differences, to identify prevention and intervention effects; however, within-group variables, such as individual differences between students, can moderate these effects (36). It is important to explore possible moderators, as these provide further information beyond the effectiveness of the program, such as sub-groups for which the program is more effective (36). The effects of the enhanced AO-PTS program have already been studied; however, there is scope for exploring the role of moderators. A study on this program investigated family functioning as a moderator, and did not find a moderation effect (37). However, as parent mental illness and family living arrangement are important risk factors for depression and anxiety in children, it is worth investigating them too.

According to ecological systems theory, the interaction between the school context, where the enhanced AO-PTS program is implemented, and the family context, could influence the effectiveness of the program for sub-groups of children (38). Considering ecological systems theory, the program may be more effective for children who have less risk factors in their family environment. Although children learn skills at school that help to protect them against depression and anxiety, they are within their family context when they are not at school. Risk factors in their family context may counteract the effects of the program or make it harder for children to apply the skills they have learnt at school. It is also possible that a moderation effect could be found in the other direction. Children who are at higher risk could improve more, simply due to there being more room for improvement.

In a randomized controlled trial of a depression prevention program for adolescents aged 13–17 in the United States, the results indicated that there was a lower rate of depressive disorder following the program; however, this was only for adolescents in the intervention group who did not have a parent with a current diagnosis of major depressive disorder (39). This suggests that individuals who did not have a parent with a current depression diagnosis benefitted more from the program (16). Another study investigated moderators of a depression prevention program for children with a family history of depression, finding that the intervention was able to reduce internalizing symptoms as well as the incidence of depressive disorder (14). In this study, parental depression status at baseline did not moderate the effects of the program. As this was an intervention involving the whole family, with both parents and children learning skills, this suggests that the involvement of the entire family may have rendered the program equally effective for children regardless of current parental depression status (14). Both of these studies were on targeted programs, and it appears that the moderating effect of parent mental illness has not been studied in a universal prevention context.

The rationale for the current study is that, as children are concurrently situated within their school and family contexts, parent mental illness and family living arrangement may moderate the intervention effects of the enhanced AO-PTS program. Both moderators are important risk factors for child mental illness (2), but have not yet been investigated in research on the enhanced AO-PTS program. This is important to better understand other factors that can influence the program's effectiveness. Furthermore, family characteristics have not been widely studied in prevention research in general, as studies tend to focus more on moderators related to the program itself, such as duration and type (29). If these family characteristics do act as moderators of the program, this could provide ways to increase the effectiveness of the program, for example, having a component of the program involving the whole family. It will also add to the literature base of prevention programs, as there have been few studies involving family context factors as moderators.

The aim of the current study was to investigate whether parent mental illness and family living arrangement moderated the effects of the enhanced AO-PTS program on depression and anxiety in children, using a cluster randomized controlled trial. The study also aimed to investigate whether parent mental illness and family living arrangement had an existing influence on children's mental health. It was hypothesized that at baseline, children who have a parent with a mental illness would score significantly higher on depression (H1a) and anxiety (H1b). It was hypothesized that at baseline, children from a higher-risk family living arrangement would score significantly higher on depression (H2a) and anxiety (H2b). It was hypothesized that parent mental illness would moderate the intervention effect—that children who did not have a parent with a mental illness would show a greater decrease in depression (H3a) and anxiety (H3b) compared to children who had a parent with a mental illness. It was hypothesized that family living arrangement would moderate the intervention effect—that children living in lower-risk family arrangements would show a greater decrease in depression (H4a) and anxiety (H4b) compared to children living in higher-risk family arrangements.

## Method

### Research design

This study was part of a longitudinal research project investigating the efficacy of the enhanced AO-PTS program. There was no protocol paper for this study. A cluster randomized controlled trial design was used. Sixteen schools were organized into matched pairs, based on school and class sizes, then the schools from each pair were randomly allocated to the intervention or control group, as described in Kennedy et al. (37). Before pre-test data collection, three of the control schools dropped out, resulting in an uneven sample of eight intervention and five control schools, with 13 schools in total. In the longitudinal project, data was collected at pre, post, 6- and 18-months. The current paper focuses on the data collected at pre-test, post-test, and 6-months only. At 18-months, due to attrition, there were only 302 cases with matching student and parent data. As this was only 32.4% of the total sample, it would be unreliable to base conclusions on this.

### Participants

The participants were primary school children aged 9–11, in years 4 and 5, who were recruited from 13 private schools in the Perth area. In the longitudinal project, of the 1,118 available children, 932 participated in the study, although not all were present at all-time points. There were 864 children present at pre-test, 835 at post-test, and 773 at the 6-month follow-up. Parents were also invited to participate, with 567 participating at pre-test. Including participants who responded at any point of the study, there was an 83.4% response rate for children, and a 62.2% response rate for parents. A CONSORT diagram can be found in the main study (Rooney et al., in preparation).

The analysis for the current study required matching student and parent data, thus the final sample included 502 children, 53.8% of the total sample. Within this sample, 46.8% (*n* = 235) were male and 53.2% (*n* = 267) were female. There were 347 children in the intervention group and 155 children in the control group. The sample size dropped to 456 children at 6-months follow-up.

### Measures

#### The children's depression inventory (CDI)

The Children's Depression Inventory [CDI; (40)] contains 27 items measuring depressive symptomology. Children select from three options which one best describes themselves in the last 2 weeks. For example, they select from “I am sad once in a while,” “I am sad many times,” or “I am sad all the time.” About half the items are reverse coded. Item scores are summed, with higher total scores indicating higher depressive symptomology. Possible scores range from 0 to 52. The CDI has consistently demonstrated good validity (41). In this study, the suicidal ideation item was removed, due to concerns from schools about it being used for children of this age. In this sample (*n* = 502), the scale demonstrated good internal consistency for the full scale, α = 0.884. The reliabilities for each subscale in this sample were: negative mood, α = 0.697; interpersonal problems, α = 0.561; ineffectiveness, α = 0.674; anhedonia, α = 0.734; and negative self-esteem, α = 0.605. Anhedonia refers to a loss of interest in activities (3).

#### Spence children's anxiety scale (SCAS)

The Spence Children's Anxiety Scale [SCAS; (42)] contains six subscales, each measuring symptoms of a separate anxiety disorder. For each of the 38 items, children select an option on a 4-point scale ranging from “never” to “always.” An example item is, “I worry about being away from my parents.” The subscale totals are summed to obtain a total score, with higher scores indicating higher levels of anxiety. The score range is 0–114. The SCAS has been found to have good validity (43).The SCAS had good internal consistency in this sample, α = 0.913. The reliabilities for each subscale in this sample were, panic attack and agoraphobia, α = 0.766; separation anxiety, α = 0.703; physical injury fears, α = 0.594; social phobia, α = 0.731; obsessive compulsive disorder, α = 0.661; and generalized anxiety disorder, α = 0.736.

#### Parent information

Parents completed a questionnaire containing demographic questions. In the current study, two variables are of interest: parent mental illness and family living arrangement. Parent mental illness was ascertained using the following questions: “has the mother received help for any mental health or psychological problems over the last 12 months?” and “has the father received help for any mental health or psychological problems over the last 12 months?” Parents were asked to select “yes” or “no” as a response to each question. They were also asked to specify which problems they had received help for, with the options being: personality problems, thought problems, attention problems, social problems, physical problems without known medical cause, aggressive behavior, anxiety/stress, sadness/depression, shyness/withdrawal, relationship problems, substance abuse, self-esteem problems, abuse, eating disorder, and other problems. They could select more than one problem. Parents disclosed their child's family living arrangement by selecting one of the following options: “mother and father together—original,” “mother and father together—blended,” “mother only,” “father only,” “mother and father separately—shared equally,” “grandparent,” “other relative,” and “legal guardian.”

### Intervention

The enhanced AO-PTS program (35) is based on cognitive-behavioral principles and aims to prevent depression and anxiety in primary school children aged 9–11 (30). The program targets attributional style, as it becomes a predictor of depression around this age (22). It also teaches children social, emotional, and cognitive skills, such as recognizing their feelings and using optimistic thinking styles (30). The program uses enactive programming, and includes activities such as building fear hierarchies and scheduling positive events (30).

The program consists of 10 one hour long modules (see Table 1) that are run in the classroom at school. Trained classroom teachers present one module each week in a whole class group setting. Teachers are trained by accredited Aussie Optimism staff and are given a program manual to use while running the program (30). Teachers are offered ongoing support from Aussie Optimism staff while they are running the program. Each child receives a workbook to use while working through the program. The program covers the learning outcomes of the Western Australian Health Curriculum (35). Intervention fidelity for this project is outlined in the main paper (Rooney et al., in preparation).

**Table 1:** The enhanced Aussie Optimism Positive Thinking program.

**Module**
1. Planning for fun activities
2. Identifying my feelings
3. Comfortable and uncomfortable feelings
4. Feelings and situations
5. Catching your thoughts
6. Being brave
7. The thought-feeling connection
8. Helpful and unhelpful thinking
9. Looking for evidence
10. Self-esteem and being brave

### Procedure

This study is part of the Enhanced Aussie Optimism Positive Thinking Project. Ethical approval was obtained from the Curtin University Human Research Ethics Committee and from the Catholic Education Department. Informed consent was obtained first from school principals and subsequently from parents and students. Schools were allocated into conditions, as described previously. Three schools dropped out of the study prior to the baseline data collection. Teachers from the intervention schools attended 1-day training workshops preparing them for the implementation of the enhanced AO-PTS program. They were also offered ongoing support while running the program, in the form of coaching from Aussie Optimism staff.

Before the program commenced, children and parents from the intervention and control groups completed the pre-test questionnaires, under the supervision of Aussie Optimism researchers. Following this, children in the intervention group participated in the enhanced AO-PTS program for 10 weeks, while children in the control group continued with their regular health program. After the program was completed, the participating children and parents filled out the post-test questionnaires. They completed the questionnaires again 6 months after this. At every time point, parents of the children in both the intervention and control groups whose questionnaires indicated clinical levels of symptomology were confidentially followed up for further assessment and referred to appropriate services through their parents.

### Data analysis

The information on parent mental illness and family living arrangement from baseline was used to test the moderation effects. This was because, due to the covariance analysis procedure, the moderator data could not be attached to particular time points. Parent mental illness was coded into two categories—“yes” and “no.” If either or both parents had sought help for a mental illness within the last 12 months, this was coded as “yes.” If neither parent had, this was coded as “no.” Family living arrangement was coded as higher-risk or lower-risk; these categories were based on previous findings in the literature (2). Lower-risk contained “mother and father together—original.” Higher-risk contained “mother and father together—blended,” “mother only,” “father only,” “mother and father separately—shared equally,” “grandparent,” “other relative,” and “legal guardian.” The higher-risk arrangements were coded as such not simply due to the arrangements themselves, but due to associated risk factors. In Australia, the prevalence of mental disorders is higher for children living in stepfamilies, blended families, single parent families, or with alternate carers (2, 15). There are a range of hypothesized reasons for this, such as: stress associated with adjusting to changes, financial difficulties, parents needing to balance work and parenting commitments, family conflict and prior circumstances contributing to the transition (11, 15, 21); all of these are possible risk factors.

The data was analyzed using covariance analysis within Generalized Linear Mixed Models (GLMM), in the SPSS (Version 22.0) GENLINMIXED procedure. This means that the effects of the intervention at post-test and 6-months were tested, controlling for baseline scores. As the data was non-independent, GLMM was appropriate, as it allows for nested data and uneven groups (44). As the outcomes were not normally distributed, robust statistics were used to compute the parameter estimates (45). Missing data was replaced at the item level using mode replacement; however, if data for an entire scale was missing, it was not replaced, but accounted for at the scale level during the analysis, as GLMM is able to account for missing data as long as data is present for one time point (44).

The analyses were run to test the effect of both moderators on the intervention effects of the program on depression and anxiety, at post-test and 6-months follow-up. The analyses controlled for pre-test scores as covariates. Each GLMM included two nominal random effects (School, Student), two nominal fixed effects (Group, Moderator), and one interval fixed effect (Pre-test Scores). The moderation effects were embodied in the Moderator × Group interactions. Follow-up analyses were run within the same GLMM analyses using least significant difference contrasts. These analyses were exploratory, as the study was not originally powered for them.

## Results

In the main study, it was found that the enhanced AO-PTS reduced the symptoms of depression at post-test and 6-months follow-up, but not at 18-months follow-up (Rooney et al., in preparation). There was also a reduction in anxiety symptoms at 6-months follow up, but not at the other time points (Rooney et al., in preparation). Following on from the intervention effects of the main study, this study tests for moderation effects.

### Parent mental illness

According to the data provided in the parent questionnaire, 80.9% of children did not have a parent with a mental illness, while 19.1% did. This represents the percentage of children who had at least one parent who sought help for a mental illness or psychological problem during the 12 months previous to baseline data collection. Of the children who had a parent with a mental illness, the highest rates were for anxiety/stress (60.5%), sadness/depression (59.7%), and relationship problems (21.8%). These percentages overlap, as some parents were experiencing more than one of the problems. The rates of other psychological problems were less than 10%.

#### Depression: children's depression inventory

Children who had a parent with a mental illness scored higher on depression at baseline by 1.740 points, 95% CI [−0.298, 3.778], *p* = 0.094; however, this difference was non-significant. There were no moderation effects of parent mental illness at post-test or 6-months follow-up (see Table 2). As the CDI contains five subscales, each reflecting a different aspect of depression, the analysis also explored the possibility that parent mental illness could influence the subscales.

**Table 2:** Testing the moderation effects of parent mental illness.

	**Post-test**	**6-months follow-up**
CDI *Total*	*F*_(1, 478)_ = 1.284, *p* = 0.258	*F*_(1, 433)_ = 0.275, *p* = 0.600
CDI *Negative mood*	*F*_(1, 478)_ = 1.932, *p* = 0.165	*F*_(1, 433)_ = 0.245, *p* = 0.621
CDI *Interpersonal behavior*	*F*_(1, 478)_ = 0.026, *p* = 0.873	*F* (1, 434) = 0.627, *p* = 0.429
CDI *Ineffectiveness*	*F*_(1, 478)_ = 0.364, *p* = 0.547	*F*_(1, 434)_ = 0.109, *p* = 0.742
CDI *Anhedonia*	*F*_(1, 478)_ = 0.327, *p* = 0.568	*F*_(1, 434)_ = 0.182, *p* = 0.669
CDI Negative self-esteem^*^	*F*_(1, 478)_ = 3.891, *p* < 0.05	*F*_(1, 434)_ = 0.003, *p* = 0.959
SCAS *Total*	*F*_(1, 471)_ = 0.016, *p* = 0.900	*F*_(1, 433)_ = 1.521, *p* = 0.218
SCAS *Agoraphobia*	*F*_(1, 478)_ = 0.408, *p* = 0.523	*F*_(1, 434)_ = 2.495, *p* = 0.115
SCAS *Separation anxiety*	*F*_(1, 478)_ = 0.115, *p* = 0.735	*F*_(1, 434)_ = 0.922, *p* = 0.337
SCAS *Physical injury*	*F*_(1, 478)_ = 0.000, *p* = 0.987	*F*_(1, 434)_ = 0.408, *p* = 0.761
SCAS *Social phobia*	*F*_(1, 477)_ = 0.850, *p* = 0.357	*F*_(1, 434)_ = 1.723, *p* = 0.190
SCAS *OCD*	*F*_(1, 474)_ = 0.959, *p* = 0.328	*F*_(1, 433)_ = 1.414, *p* = 0.235
SCAS *GAD*	*F*_(1, 476)_ = 1.581, *p* = 0.209	*F*_(1, 434)_ = 0.455, *p* = 0.501

*Statistical significance*:

* *p < 0.05*.

At post-test, parent mental illness moderated the effects of the program on the CDI subscale *negative self-esteem*. The follow-up analysis revealed that among children who had a parent with a mental illness, there was no significant difference between the intervention and control groups at post-test, after controlling for baseline scores. However, there was an effect found among children who did not have a parent with a mental illness, where the intervention group scored significantly lower than the control group by 0.155 points, 95% CI [0.038, 0.272], *p* < 0.01, after controlling for baseline differences.

At post-test, parent mental illness did not moderate the effects of the program on the subscales: *negative mood, interpersonal behavior, ineffectiveness, or anhedonia*. At 6-months follow-up, parent mental illness did not moderate the effects of the program on any of the CDI subscales.

#### Anxiety: spence children's anxiety scale

At baseline, children who had a parent with a mental illness scored higher on the SCAS by 2.433 points, 95% CI [−1.211, 6.078], *p* = 0.190, than children who did not. This, however, was not a significant difference. There were no moderation effects at post-test, or 6-months follow-up. Each subscale of the SCAS measures the symptomology of a different anxiety disorder, so the effects of parent mental illness on each of these was explored. The anxiety disorders are agoraphobia, separation anxiety, physical injury, social phobia, obsessive compulsive disorder (OCD), and generalized anxiety disorder (GAD). OCD is no longer classified as an anxiety disorder; however it was at the time of the study (45). At post-test, parent mental illness did not moderate the effects of the program on any of the subscales of the SCAS. These were also no moderation effects at 6-months follow-up (see Table 2).

### Family living arrangement

Parent reported data indicated that 81.1% of children lived within a lower-risk family living arrangement and 18.9% lived within a higher-risk arrangement (see Table 3).

**Table 3:** Children in each family living arrangement.

**Family living arrangement**	**Percentage of children (%)**
Mother and father together—original	81.1
Mother and father together—blended	2.9
Mother only	8.4
Father only	0.5
Mother and father separately—shared equally	6.4
Grandparent	0.4
Other relative	0
Legal guardian	0.2

#### Depression: children's depression inventory

At baseline, children from a higher-risk family living arrangement scored significantly higher in depression than children from a lower-risk family arrangement, by 2.370 points, 95% CI [1.505, 3.236], *p* < 0.001. Family living arrangement did not moderate the effect of the enhanced AO-PTS program on depression at post-test or 6-months follow-up (see Table 4).

**Table 4:** Testing the moderation effects of family living arrangement.

	**Post-test**	**6-months follow-up**
CDI *Total*	*F*_(1, 502)_ = 1.569, *p* = 0.211	*F*_(1, 455)_ = 1.117, *p* = 0.291
CDI *Negative mood*	*F*_(1, 497)_ = 0.068, *p* = 0.795	*F*_(1, 450)_ = 0.074, *p* = 0.786
CDI Interpersonal behaviour^*^	*F*_(1, 497)_ = 6.885, *p* < 0.01	*F*_(1, 451)_ = 0.081, *p* = 0.776
CDI *Ineffectiveness*	*F*_(1, 497)_ = 1.352, *p* = 0.246	*F*_(1, 451)_ = 0.001, *p* = 0.981
CDI *Anhedonia*	*F*_(1, 497)_ = 2.751, *p* = 0.098	*F*_(1, 451)_ = 3.804, *p* = 0.052
CDI N*egative self-esteem*	*F*_(1, 497)_ = 2.956, *p* = 0.086	*F*_(1, 451)_ = 0.310, *p* = 0.578
SCAS *Total*	*F*_(1, 497)_ = 0.014, *p* = 0.907	*F*_(1, 450)_ = 0.465, *p* = 0.496
SCAS *Agoraphobia*	*F*_(1, 497)_ = 0.011, *p* = 0.916	*F*_(1, 451)_ = 0.007, *p* = 0.934
SCAS *Separation anxiety*	*F*_(1, 497)_ = 0.333, *p* = 0.564	*F*_(1, 451)_ = 0.364, *p* = 0.547
SCAS *Physical injury*	*F*_(1, 497)_ = 0.020, *p* = 0.888	*F*_(1, 451)_ = 2.430, *p* = 0.120
SCAS *Social phobia*	*F*_(1, 496)_ = 0.089, *p* = 0.765	*F*_(1, 451)_ = 1.211, *p* = 0.272
SCAS *OCD*	*F*_(1, 493)_ = 0.001, *p* = 0.972	*F*_(1, 450)_ = 0.009, *p* = 0.923
SCAS *GAD*	*F*_(1, 495)_ = 0.003, *p* = 0.953	*F*_(1, 451)_ = 0.097, *p* = 0.756

Statistical significance: ^**^p < 0.01.

At post-test, family living arrangement did not moderate the effects of the program on the subscales: *negative mood, ineffectiveness, anhedonia, and negative self-esteem*. There was a significant interaction between group and family living arrangement for *interpersonal behavior*. Within the intervention group, higher-risk students scored 0.265 points higher, 95% CI [0.058, 0.472], *p* < 0.05 on interpersonal behavior. However, this was not evidence of family living arrangement moderating the intervention effect, and thus was not relevant to the hypothesis testing. At 6-months follow-up, family living arrangement did not moderate the effects of the program on any of the CDI subscales.

#### Anxiety: spence children's anxiety scale

At baseline, children from a higher-risk family living arrangement scored higher in anxiety than children from a lower-risk family arrangement, by 3.286 points, 95% CI [−0.097, 6.669], *p* = 0.057, however this was not a significant difference. There were no moderation effects at post-test or at 6-months follow up. Family living arrangement did not moderate the effects of the program on any of the SCAS subscales at post-test or at 6-months follow-up (see Table 4).

## Discussion

A study on the longitudinal project has already found results indicating that the enhanced AO-PTS program contributed to significant reductions in depression at post-test and 6-months follow-up, and and anxiety at 6-months follow-up (Rooney et al., in preparation). This study explored the role of family-based contextual factors that might influence the effectiveness of the program, specifically, parent mental illness and family living arrangement.

Before testing for moderation effects, the main effects of parent mental illness and family living arrangement were tested, to establish whether they had an existing influence on depression and anxiety at baseline, before children had participated in the program.

Children who had a parent with a mental illness did not score significantly higher on depression (H1a) and anxiety (H1b) at baseline than children who did not; thus, the hypothesis was not supported. This result is unexpected, as studies have consistently found that parent mental illness is a risk factor for child depression (11). A possible reason for this finding may have been that the self-report data led to an underestimation of the prevalence of mental illness in this sample. However, this does not seem likely, as the rate in this sample was very similar to Australian rates, where 21% of children live in a family where at least one parent has a mental illness (15). Another explanation may be that, since the question asked if parents had sought help for a mental illness over the past year, the help that they had received had been effective and their mental illness no longer had an impact on their child at the time of the study. Family history is a risk factor whether or not the parent is currently experiencing a psychological problem (3); however, there may be a greater impact if the problem is current (39).

Children from a higher-risk family living arrangement scored significantly higher on depression, with a difference of 2.37 points in the CDI, which supported the hypothesis (H2a). This aligned with previous research results, which indicate higher rates of mental illness among children in stepfamilies, single parent families, and with alternate carers (2, 15). This result suggests that the family context should be taken into account in the prevention of depression, and that families, especially those who may be going through a time of transition, should be provided with support. However, children from a higher-risk family living arrangement did not score significantly higher in anxiety (H2b).It is unusual that a main effect was found for depression, but not anxiety, as the family context can influence children's mental health overall, not just depression (15). It is possible that the effect of family living arrangement on different anxiety disorders contributed to this lack of a significant result. It has been found that although there is a general trend of lower rates for children living with biological or adoptive parents, the rates of prevalence differ according to specific anxiety disorders (2).

Parent mental illness and family living arrangement did not moderate the effects of the program on depression or anxiety, as measured with the full scale CDI and SCAS, thus the hypotheses were not confirmed (H3a, H3b, H4a, H4b). This suggests that there were no subgroup effects of the program based on family risk factors. The results did not align with Bronfenbrenner's (12) theory, which would suggest that the program would be more effective for children who live in a lower-risk family environment.

A possible explanation for the lack of moderation effects for family living arrangement is that there were two opposite influences counteracting each other. In this study, this is particularly relevant for the results on depression. The first influence is that already discussed—a higher-risk family environment rendering it more challenging for children to develop the skills they learn during the program (16), as suggested by ecological systems theory (12). The second influence is that of elevated baseline scores, with children in a higher-risk family living arrangement scoring significantly higher on depressive symptoms at baseline. It follows that, due to their scores being already higher before the study, there was more room for improvement. This is supported by the literature on universal and targeted programs, where larger effect sizes are found for groups of children who are at risk or have already elevated symptoms (24). It has been argued that greater improvements are found in higher-risk groups because there is more capacity for improvement due to higher baseline scores (29). Thus, an alternate explanation for the findings is that both these influences were present during this study, and accounted for the lack of moderation effects for family living arrangement.

However, the lack of moderation effects is a positive outcome for the enhanced AO-PTS program and its efficacy, as it indicates the benefits of it being run as a universal prevention program. It is effective in reducing depressive and anxiety symptoms for children regardless of whether they are at-risk or not, in terms of family risk factors.

As each subscale of the CDI and the SCAS represents a different symptom or type of depression and anxiety, the analysis also tested whether the family risk factors moderated the effects of the program on any of these. It was found that at post-test, parent mental illness moderated the effects of the program on negative self-esteem, which is a subscale of the CDI. There was no effect for children who had a parent with a mental illness, but for children who did not have a parent with a mental illness, the intervention group children improved significantly compared to the control group children. This aligned with the hypothesis (H3a); however, only for one aspect of depression. Even so, this suggests that parent mental illness does have the capacity to affect the results of prevention programs. This result is similar to that found in a study of another depression prevention program (39). Furthermore, this finding shows that it is worth investigating family risk factors as moderators in universal prevention programs.

It is of interest that negative self-esteem appeared to be the aspect of depression that was most influenced by parent mental illness in this study. Self-esteem is a concept that is specifically addressed in the AO-PTS program (35), and is a protective factor against depression, as explained in the self-esteem buffering hypothesis (46). This hypothesis involves the concept of dependency, which refers to an individual's reliance on others for their well-being, and the related fear of rejection from others (46). A study on the self-esteem buffering hypothesis was conducted with children whose parents had a current or past diagnosis of major depressive disorder. It was found that among children with low self-esteem, high dependency was associated with increased depressive symptoms after negative life events. However, this association was not present for children with high self-esteem, illustrating the buffering role of self-esteem (46). The results of the current study show that children who had a parent with a mental illness did not improve in negative self-esteem at post-test. This may simply be because self-esteem is the final module in the program, and that they needed more time to integrate this information than children who did not have a parent with a mental illness. To further assist learning on self-esteem, perhaps the module could be accompanied with an activity that children can complete with their parents, thus involving the whole family.

### Limitations

This study was limited in that information on parent mental illness and family living arrangement was not available for all the participants, and the results therefore may not be reflective of the entire sample in the larger project. There was data available for 53.8% of participants to test the effects from baseline to post-test, and 48.9% from baseline to 6-months follow-up. These low percentages are problematic as the study focused on the family context, and parents who had a mental health problem would be less likely to complete the questionnaire. A related limitation is that the analyses can only be seen as exploratory, as the study was not powered for them originally. Due to their only being data for about half the children and parents, the analysis is lacking in power, and therefore conclusions are not representative of the whole sample of children who participated. It is also a limitation that data on the moderators was taken from baseline, as parent mental illness and family living arrangement can change over time. Taking the information from one time point may not have accurately represented the situation over the course of the study.

In addition, children were all from private schools, and may not have been representative of the population in terms of both symptomology and family characteristics, decreasing the external validity of the study. Of the 13 schools, 11 of these had an above average ICSEA score, which is a measure of students' socio-educational backgrounds (47). However, the schools did represent a wide range of locations, with schools spread across the inner and outer suburbs of Perth in all directions. Another limitation was that information was not collected on the further help that students were able to access after the clinical interview, and therefore this could not be separated out as another possible influence on the results.

As parent mental illness was self-reported, the validity of this information is uncertain, as parents who indicated that they did have a mental health problem may not have actually received a clinical diagnosis. Parents may also have had a mental health problem and been unaware of it. The question itself also presented a limitation. It asked if parents had received help for any mental health or psychological problem; however, many individuals with a mental health problem do not seek help. Thus, there may have been parents who did have a mental illness but were not identified due to the wording of the question. In addition, data was collected on parent mental illness within the past year only; however, incidences before this could already have affected children, especially as this is also a biological risk factor (3). For future research, it would be better to word this question differently. The question on family living arrangement was also limited in scope, as there were other possible family living arrangements that were not included in the questionnaire. As mentioned previously, another limitation is that the information on parent mental illness and family living arrangement was from baseline, and both of these could have changed over the course of the study.

It is possible that having only two levels within each moderator did not capture what was happening. Instead of grouping all mental illnesses together, it may have been better to explore the effects of specific mental illnesses, for example, whether having a parent with an anxiety disorder influenced the effect of the program on anxiety symptoms. Research has shown that different mental illnesses affect children in various ways (17). It would also have been beneficial to look at each of the eight family living arrangements separately, instead of grouping them into two categories. However, this was not possible, because there were not enough children in each category to do this. The reliabilities for four of the depression subscales and two of the anxiety subscales were below 0.70, which presents a limitation as the reliability of the measurements of these specific aspects of depression and anxiety are uncertain. These lower values might have been due to not being able to use the data from the entire sample, because of the low rate of matching student and parent data.

### Future research

Future research could explore the influence of parent mental illness and family living arrangement on the effects of universal programs other than Aussie Optimism. Research could also address the role of other family characteristics, such as socioeconomic status and parent-child relationship quality (3). Research could look at interactions between family factors, and whether combinations of factors, rather than factors alone, influence the effectiveness of the program. This would better reflect the real world context, where multiple factors interact together to influence mental health outcomes. It could also look at school-based risk factors, such as social inclusion and school environment (3).

Future research could also address parent and teacher reported outcomes, in addition to child reports. Triangulation of sources is especially valuable when children are at a primary school age, as children may not yet have the level of self-awareness to identify their own symptoms of depression and anxiety (3). An additional qualitative component would be beneficial, as this could capture perspectives about the influence of the family context that are unable to be captured by quantitative data.

In summary, the finding that parent mental illness and family living arrangement did not moderate the effects of the enhanced AO-PTS program on depressive and anxiety symptoms suggests that the program is equally effective for a whole range of children, and is suitable for use as a universal program. The study also contributes to the evidence base that family risk factors are related to children's mental health, specifically the finding on the differences in depressive symptoms based on family living arrangement. Finally, the moderation effect of parent mental illness on negative self-esteem, an aspect of depression, suggests that it is worth conducting further research on the influence that contextual factors may have on the efficacy of prevention programs.

## Ethics statement

This study was carried out in accordance with the recommendations of the Curtin University Human Research Ethics Committee with written informed consent from all subjects. All subjects, as well as their parents, gave written informed consent in accordance with the Australian Psychological Society Code of Ethics. The protocol was approved by the Curtin University Human Ethics Committee, as well as the Catholic Education Department.

## Author contributions

MC conceptualized, conducted the analyses, and was principal author of publication. RR was the Principal Investigator of the grant and wider research project. She helped to conceptualize and assisted with writing the publication. RK was a Chief Investigator of the research project and provided assistance with conceptualization as well as expertise in statistical analyses. SH was a Chief Investigator of the research project and provided assistance with conceptualization of the publication. NB contributed to the writing of the manuscript.

### Conflict of interest statement

The authors declare that the research was conducted in the absence of any commercial or financial relationships that could be construed as a potential conflict of interest.
